# Prognostic value of the Naples prognostic score in patients with intrahepatic cholangiocarcinoma after hepatectomy

**DOI:** 10.1186/s12885-024-12502-4

**Published:** 2024-06-14

**Authors:** Cheng-Fei Du, Zhen-Yu Gao, Zhu-Ding Xu, Zheng-Kang Fang, Zi-Chen Yu, Zhe-Jin Shi, Kai-Di Wang, Wen-Feng Lu, Xiao-Kun Huang, Lei Jin, Tian-Wei Fu, Guo-Liang Shen, Jun-Wei Liu, Cheng-Wu Zhang, Dong-Sheng Huang, Lei Liang

**Affiliations:** 1 Department of General Surgery, Cancer Center, Hepatobiliary & Pancreatic Surgery and Minimally Invasive Surgery, Zhejiang Provincial People’s Hospital (Affiliated People’s Hospital), Hangzhou Medical College, Hangzhou, 310014 Zhejiang China; 2 Department of the Second School of Clinical Medicine, Zhejiang Chinese Medical University, Hangzhou, China; 3 Department of Hepatobiliary Surgery, Eastern Hepatobiliary Surgery Hospital, Second Military Medical University (Navy Medical University), Shanghai, China

**Keywords:** Naples prognostic score, Intrahepatic cholangiocarcinoma, Inflammation, Nutritional status, Prognosis

## Abstract

**Background:**

The Naples Prognostic Score (NPS), integrating inflammatory and nutritional biomarkers, has been reported to be associated with the prognosis of various malignancies, but there is no report on intrahepatic cholangiocarcinoma (ICC). This study aimed to explore the prognostic value of NPS in patients with ICC.

**Methods:**

Patients with ICC after hepatectomy were collected, and divided into three groups. The prognosis factors were determined by Cox regression analysis. Predictive efficacy was evaluated by the time-dependent receiver operating characteristic (ROC) curves.

**Results:**

A total of 174 patients were included (Group 1: 33 (19.0%) patients; Group 2: 83 (47.7%) patients; and Group 3: 58 (33.3%) patients). The baseline characteristics showed the higher the NPS, the higher the proportion of patients with cirrhosis and Child-Pugh B, and more advanced tumors. The Kaplan-Meier curves reflect higher NPS were associated with poor survival. Multivariable analysis showed NPS was an independent risk factor of overall survival (NPS group 2 vs. 1: HR = 1.671, 95% CI: 1.022–3.027, *p* = 0.009; NPS group 3 vs. 1: HR = 2.208, 95% CI: 1.259–4.780, *p* = 0.007) and recurrence-free survival (NPS group 2 vs. 1: HR = 1.506, 95% CI: 1.184–3.498, *p* = 0.010; NPS group 3 vs. 1: HR = 2.141, 95% CI: 2.519–4.087, *P* = 0.001). The time ROC indicated NPS was superior to other models in predicting prognosis.

**Conclusions:**

NPS is a simple and effective tool for predicting the long-term survival of patients with ICC after hepatectomy. Patients with high NPS require close follow-up, and improving NPS may prolong the survival time.

**Supplementary Information:**

The online version contains supplementary material available at 10.1186/s12885-024-12502-4.

## Introduction

Intrahepatic cholangiocarcinoma (ICC) is a malignant tumor originating from the epithelial cells of the intrahepatic bile ducts, accounting for up to 15% of primary liver cancer, with an increasing morbidity and mortality [1]. Complete surgical resection involving a formal liver resection and portal lymphadenectomy is still recognized as the only potential curative treatment for patients with ICC [2]. However, the 5-year survival rate after curative-intent resection is still unsatisfactory, only about 20–35% [3–5]. Local and/or distant recurrence after surgery is the main reason that impedes a cure in patients with resectable ICC. Therefore, it is crucial to explore valuable prognostic indicators to identify patients with a high risk of recurrence and guide anti-recurrence therapy.

In the past decade, the involvement of inflammation in the development and progression of cancer has been well-established, particularly in facilitating tumor cell proliferation and metastasis. The majority of ICC patients evolve in the setting of chronic inflammation, such as biliary stone disease or hepatitis B virus (HBV) infection [5–8]. Previously, it has been demonstrated that various inflammatory indicators in the serum are associated with the prognosis of ICC, such as the neutrophil-to-lymphocyte ratio (NLR) [9], the lymphocyte-to-monocyte ratio (LMR) [9], the platelet‑to‑lymphocyte ratio (PLR) [10], the systemic inflammation score (SIS) [11]. Meanwhile, the liver is also an important organ involved in nutrient metabolism and protein production. Chronic inflammation often leads to liver function damage, even cirrhosis. Malnutrition is also associated with inflammation, oxidative stress, and altering metabolic state, thereby affecting tumor progression [12]. Previous studies also have shown that nutritional status was also associated with poor survival rates, such as prognostic nutritional index (PNI) [13, 14] and albumin–bilirubin (ALBI) [15]. However, the prognostic value of these aforementioned variables remains controversial, which may be due to significant collinearity among variables (such as NLR and LMR). Therefore, there is an urgent requirement for a comprehensive prognostic model that incorporates indicators associated with inflammation and nutrition.

The Naples Prognostic Score (NPS), proposed by Galizia et al [16], is a new prognostic index integrating inflammatory with nutritional biomarkers, including serum albumin, total cholesterol levels, the NLR, and LMR. The NPS has been reported to be associated with the prognosis of various tumors [17–22], but there is no report in ICC. This study aims to explore the prognostic value of NPS in patients with ICC after hepatectomy and compare its predictive ability with other inflammatory and nutritional indicators.

## Materials and methods

### Patients

All patients who underwent curative surgical (R0) resection were pathologically confirmed to have ICC from Jan. 2014 to Dec. 2020 at Zhejiang Provincial People’s Hospital and were considered for inclusion. R0 resection is defined as complete resection of the tumor with negative microscopic margins. The exclusion criteria were as follows: (1) age < 18 years old, (2) preoperative antitumor therapy, (3) inflammatory diseases or other infections in the month before surgery (including arthritis, glomerulonephritis, pneumonia, nervous system infection, acute cholecystitis or pancreatitis, etc.), (4) received preoperative anti-infective or nutritional supportive treatments, (5) patients with tumor recurrence in 30 days or died in 90 days after surgery, (5) combined ICC and hepatocellular carcinoma, and (6) incomplete data records. All patients included in the study had obtained informed consent before surgery and agreed to have their data stored and used in the research. This study was consistent with the Declaration of Helsinki and approved by the Institutional Review Board at Zhejiang Provincial People’s Hospital.

### Preoperative NPS and other scoring systems

The definition and calculation formula of NPS and other scoring systems (NLR, LMR, SIS, PNI, and ALBI) are all based on previous research reports. NPS [16] = serum albumin (< 4.0 g/dL = 1, ≥ 4.0 g/dL = 0) + total cholesterol concentrations (< 180 mg/dL = 1, ≥ 180 mg/dL = 0) + LMR (< 4.44 = 1, ≥ 4.44 = 0) + NLR (< 2.96 = 0, ≥ 2.96 = 1). All patients, then, were divided into 3 groups: Group 1 (NPS = 0); Group 2 (NPS = 1 or 2), and Group 3 (NPS = 3 or 4), respectively (Supplement Fig. 1). SIS [11] was calculated as (serum albumin ≥ 4 g/dL and LMR ≥ 4.44 = 0, either serum albumin < 4.0 g/dL or LMR < 4.44 = 1, both serum albumin < 4 g/dL and LMR < 4.44 = 2). PNI [14] was calculated as serum albumin (g/L) + 0.005×total lymphocyte count (10^9^/L). ALBI [15] was calculated as [log10bilirubin (mmol/L) * 0.66] + [albumin (g/L) * −0.085]. According to the results of the time-dependent ROC, the cut-off value of PLR, PNI, and ALBI was set at 200, 47, and − 2.70, respectively.

**Fig. 1:**
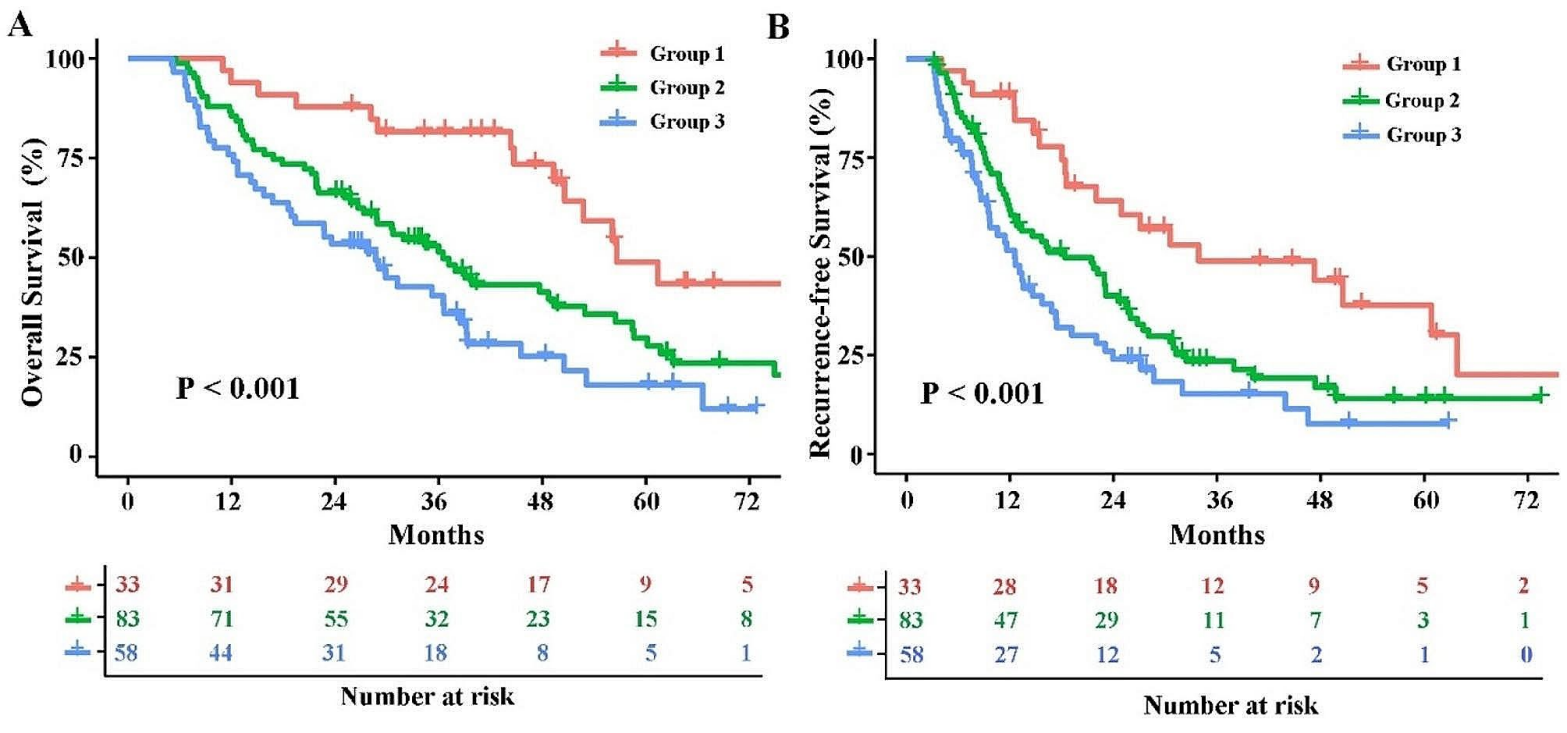
The K-M curves comparisons of overall survival and recurrence free survival among each group (calculated by Log rank test). Group 1 (NPS = 0); Group 2 (NPS = 1 or 2), and Group 3 (NPS = 3 or 4), respectively. (**A**) Overall survival, (**B**) Recurrence-free survival

### Study variables and followed-up

Patient information was retrospectively gathered from the hospital case system. These variables included sex, age (> 65 vs. ≤ 65 years), co-morbid illness (consists of cardiovascular disease, renal dysfunction history, obesity, and diabetes mellitus), physical status (PS), American Society of Anesthesiologists (ASA) score, HBV, the presence of cirrhosis, Child-Pugh (A/B), serum CEA and CA19-9 level, serum alanine aminotransferase (ALT) and aspartate aminotransferase (AST), tumor size, number of tumors, microvascular invasion (MVI), tumor differentiation, resection margin (< 1 vs. ≥ 1 cm), type of hepatic resection (anatomical vs. non-anatomical), lymph node metastasis, intraoperative blood loss (> 400 vs.≤ 400 mL), perioperative blood transfusion, and postoperative adjuvant chemotherapy. Adjuvant chemotherapy included oral capecitabine or intravenous infusion of gemcitabine and oxaliplatin. The postoperative morbidities included post hepatectomy liver failure (PHLF), bleeding, blood transfusion, bile leakage, surgical site infection, abdominal hemorrhage, pneumonia, pleural effusion, ascites, and other conditions (such as acute pancreatitis, acute cholangitis, and cardiocerebrovascular accidents). The definition of PHLF was determined according to the International Study Group of Liver Surgery (ISGLS) definition in 2011 [23]. The Clavien-Dindo system was used to classify the postoperative morbidities: major morbidity was defined as grades III-IV, while minor morbidity was graded I-II [24]. Postoperative morbidities were collected during the patient’s hospitalization.

Patients were followed every 3 months for the first 2 years and every 6 months after 2 years. At each of the follow–up visits, serum tumor biomarkers (CA 19–9 and CEA), and abdominal ultrasound were detected. Chest CT and abdominal enhanced CT or MRI are performed every 3 months or when there is suspicion of tumor recurrence. Treatments of recurrence were tailored according to the tumor burden and general condition of the patient, including radical resection, chemotherapy, target therapy or immunotherapy. OS and RFS were calculated from the date of hepatectomy until the date of the most recent follow-up or death of the patient and as clinical evidence of tumor recurrence, respectively.

### Statistical methods

Categorical variables were expressed using frequencies and percentages and compared by *X*^2^ test or Fisher exact tests, as appropriate. The survival curves were estimated using the Kaplan-Meier method and compared using the log‐rank test. Variable with *P* < 0.1 in the univariate Cox -regression analysis was included in the multivariate prognostic analysis. The predictive ability of each scoring system was evaluated based on the time-dependent receiver operating characteristic (ROC) curves and calculating the area under the curve (AUC). The comparison of AUC differences between different variables adopts the DeLong test. *P* < 0.05 was set as a statistical difference. The study was statistically analyzed using R 4.3.1 software (http://www.r-project.org/).

## Results

### Baseline characteristics

A total of 174 patients with ICC were enrolled, including 106 males and 68 females. Among them, 51 (29.3%) patients had HBV infection, and 47 (27.0%) patients had cirrhosis. In addition, 83 (47.7%) patients had tumors in AJCC 8th TNM stage II or higher. Moreover, 103 (59.2%) patients underwent lymph node resection, with a median number of 8 (range 1–23). Among the patients who underwent lymph node resection, 41 patients were found to have positive lymph node metastasis. After surgery, 81(46.6%) patients received adjuvant chemotherapy. Then, all patients were stratified into 3 groups according to preoperative NPS: Group 1: 33 (19.0%) patients, Group 2: 83 (47.7%) patients, and Group 3: 58 (33.3%) patients, respectively (Table 1). By comparing the clinical characteristics of each group, the results indicated that the higher the NPS score, the higher the proportion of patients with cirrhosis and poor liver function (Child-Pugh B). In addition, the higher the NPS score, the more advanced the tumor is (all *P* < 0.05).

**Table 1:** Comparisons of clinical characteristics among the three groups according to the Naples prognostic score

Variable (*N*, %)	Group 1 (*n* = 33)	Group 2 (*n* = 83)	Group 3 (*n* = 58)	*P* Value
Sex, Male	19 (57.6)	47 (56.6)	40 (69.0)	0.305
Age, > 65 years	15 (45.5)	39 (47.0)	27 (46.6)	0.989
Co-morbid illness	8 (24.2)	15 (18.1)	14 (24.1)	0.617
ASA, > 2	9 (27.3)	20 (24.1)	16 (27.6)	0.878
Performance status, ≥ 1	15 (45.5)	44 (53.0)	38 (65.5)	0.141
HBV (+)	8 (24.2)	25 (30.5)	18 (31.0)	0.762
PLT, ≥ 100*10^9^/L	30 (90.9)	79 (95.2)	53 (91.4)	0.585
ALT, > 40 IU/L	12 (36.4)	23 (27.7)	21 (36.2)	0.483
AST, > 40 IU/L	11 (33.3)	20 (24.1)	23 (39.7)	0.138
Child-Pugh, A/B	31 (93.9)/2 (6.1)	70 (84.3)/13 (15.7)	42 (72.4)/16 (27.6)	0.028
Cirrhosis	7 (21.2)	19 (22.9)	21 (36.2)	0.152
CEA, > 10 ug/L	4 (12.1)	12 (14.5)	14 (24.1)	0.224
CA19-9, > 37 ug/L	15 (45.5)	54 (65.1)	40 (69.0)	0.068
AJCC 8th TNM stage, ≥ II	7 (21.2)	43 (51.8)	33 (56.9)	0.003
Maximum tumor size, > 5 cm	7(21.2)	29 (34.9)	27 (46.6)	0.051
Tumors number, ≥ 2	1 (3.0)	9 (10.8)	12 (20.7)	0.041
Resection margin, > 1 cm	19 (57.6)	46 (55.4)	32 (55.2)	0.972
Anatomical resection	28 (84.8)	65 (78.3)	42 (72.4)	0.383
MVI (+)	6 (18.2)	32 (38.6)	23 (39.7)	0.106
lymph node metastasis	4 (12.1)	18 (21.7)	19 (32.8)	0.071
Differentiation, moderate-poor	29 (87.9)	76 (91.6)	52 (89.7)	0.820
Blood loss, > 400 ml	16 (48.5)	37 (44.6)	28 (48.3)	0.883
Blood transfusion	14 (42.4)	39 (47.0)	29 (50.0)	0.784
Operation time, > 300 min	12 (36.4)	18 (21.7)	22 (37.9)	0.077
Adjuvant chemotherapy	14 (42.4)	44 (53.0)	23 (39.7)	0.256
NLR,>2.96/ ≤2.96	2 (6.1)/31 (93.9)	20 (24.1)/63 (75.9)	45 (77.6)/13 (22.4)	0.001
LMR, ≤ 4.4/ >4.4	3 (9.1)/30 (90.9)	28 (33.7)/55 (66.3)	55 (94.8)/3 (5.2)	0.001
PLR, ≥ 300/<300	3 (9.1)/ 30 (90.9)	12 (14.5)/ 71 (85.5)	16 (27.6)/ 42 (72.4)	0.047
SIS, 0	30 (90.9)	22 (26.5)	1 (3.0)	0.001
1	2 (6.1)	53 (63.9)	13 (21.1)	
2	1 (3.0)	8 (9.6)	44 (75.9)	
PNI, ≤ 47/ >47	0 (0)/33 (100.0)	35 (42.2)/48 (57.8)	49 (84.5)/9 (15.5)	0.001
ALBI, ≥ −2.70/<−2.70	1 (3.0)/32 (97.0)	7 (8.4)/76 (91.6)	28 (48.3)/30 (51.7)	0.001

Abbreviations: ASA: Physical Status classification system; HBV: hepatitis B virus; PLT: platelet count; ALT: alanine aminotransferase; AST: aspartate aminotransferase; CEA: carcinoembryonic antigen; CA19-9: Carbohydrate Atigen19-9; MVI: microvascular invasion; NLR: neutrophil to lymphocyte ratio; LMR: the lymphocyte to monocyte ratio; SIS: systemic inflammation score; PNI: prognostic nutritional index; ALBI: albumin–bilirubin

### Postoperative morbidity

Postoperative morbidities were collected during the patient’s hospitalization (Table 2). The incidence of overall morbidity was 39.7% (Group 1: 27.3% vs. Group 2, 36.1% vs. Group 3: 51.7%, *P* = 0.048, respectively). Of these, 32.8% was minor morbidity (Group 1: 18.2% vs. Group 2, 30.1% vs. Group 3: 44.8%, *P* = 0.048) and 6.9% were major morbidity (Group 1: 9.1% vs. Group 2, 6.0% vs. Group 3: 6.9%, *P* = 0.841). The results showed high grade of NPS was significantly associated with postoperative morbidity, especially for minor morbidity (*P* = 0.026). In detail, although there was no statistical difference, a lower grade of NPS is superior to a higher grade of NPS in reducing PHLF, bile leakage, surgical site infection, and pleural effusion. No patient died during the patient’s hospitalization.

**Table 2:** Comparisons of postoperative morbidity among the three groups according to the Naples prognostic score

Variable (*N*, %)	Group 0 (*n* = 33)	Group 1 (*n* = 83)	Group 2 (*n* = 58)	*P* value
Overall morbidity	9 (27.3)	30 (36.1)	30 (51.7)	0.048
Clavien-Dindo, I-II	6 (18.2)	25 (30.1)	26 (44.8)	0.026
III-IV	3 (9.1)	5 (6.0)	4 (6.9)	0.841
PHLF	2 (6.1)	7 (8.4)	6 (10.3)	0.780
Abdominal hemorrhage	1 (3.0)	0 (0)	3 (5.2)	0.125
Bile leakage	2 (6.1)	2 (2.4)	4 (6.9)	0.414
Surgical site infection	4 (12.1)	4 (4.8)	7 (12.1)	0.233
Pneumonia	0 (9.6)	8 (9.6)	4 (6.9)	0.181
Pleural effusion	5 (15.2)	16 (19.3)	15 (25.9)	0.435
Ascites	3 (9.1)	9 (10.8)	15 (25.9)	0.028
Others^#^	1 (3.0)	4 (4.8)	6 (10.3)	0.286

PHLF: post hepatectomy liver failure. ^#^Others include acute pancreatitis; acute cholangitis; and cardiocerebrovascular accidents

### Overall survival and recurrence-free survival

After a median of 34.0 months of follow-up, death and recurrence were observed in 113 (64.9%) and 127 (73.0%) patients. For the entire cohort, the 1-, 3-, and 5-year OS and RFS were 82%, 52% and 30%, and 60%, 26% and 17%. The 1-, 3-, and 5- years OS among each NPS group were 94%, 82%, and 49% in Group 1, 84%, 50%, and 30% in Group 2, and 71%, 36% and 18% in Group 3, respectively (Fig. 1A). Accordingly, the 1-, 3-, and 5- years RFS among each NPS group were 84%, 49%, and 38% in Group 1, 58%, 23%, and 14% in Group 2, 48%, 15%, and 8% in Group 3, respectively (Fig. 1B). The K-M curves showed that a higher grade of NPS was significantly associated with poorer OS and RFS (both *P* < 0.001).

### Univariable and multivariable Cox-regression analyses

Tables 3 and 4 show the results of the Cox regression analysis. In the multivariable analyses, NLR, LMR, SIS, PNI, and ALBI were analyzed separately with other variables to avoid covariance with NPS. Multivariable analysis showed NPS was an independent risk factor of OS (NPS group 2 vs. 1: HR = 1.671, 95% CI: 1.022–3.027, *P* = 0.009; NPS group 3 vs. 1: HR = 2.208, 95% CI: 1.259–4.780, *P* = 0.007) and RFS (NPS group 2 vs. 1: HR = 1.506, 95% CI: 1.184–3.498, *P* = 0.010; NPS group 3 vs. 1: HR = 2.141, 95% CI: 2.519–4.087, *P* = 0.001). Moreover, the results of multivariable analyses showed that SIS and PNI were independent risk factors for OS and RFS, while NLR and LMR were not independent risk factors for both OS and RFS. Additionally, ALBI was an independent risk factor for OS, but not for RFS.

**Table 3:** Univariable and multivariable Cox regression analyses of prognostic factors associated with overall survival for patients with intrahepatic cholangiocarcinoma after hepatectomy

Variables	UV HR (95% CI)	UV *P*	MV HR (95% CI)	MV *P*^#^
Sex, Male vs. Female	1.321 (0.907–1.922)	0.145		
Age, > 65 vs.≤ 65 years	1.242 (0.863–1.786)	0.243		
Co-morbid illness, yes vs. no	1.014 (0.487–1.230)	0.279		
ASA,>2 vs. ≤ 2	1.174 (0.656–2.346)	0.326		
Performance status, ≥ 1 vs. <1	1.157 (0.445–3.219)	0.401		
HBV, yes vs. no	1.191 (0.794–1.786)	0.397		
PLT, ≥ 100*10^9^/L vs. < 100*10^9^/L	1.091 (0.508–2.344)	0.822		
ALT, > 40 vs. ≤ 40 IU/L	1.018 (0.728–1.604)	0.700		
AST, > 40 vs. ≤ 40 IU/L	1.110 (0.756–1.631)	0.594		
Child-Pugh, B vs. A	1.171 (0.852–1.823)	0.145		
Cirrhosis, yes vs. no	0.972 (0.643–1.470)	0.894		
CEA, > 10 ug/L vs. ≤10 ug/L	1.302 (0.887–1.912)	0.177		
CA19-9, > 37 ug/L vs. ≤37 ug/L	2.343 (1.517–3.618)	0.001	1.117 (1.104–3.215)	0.018
Maximum tumor size,> 5 vs. ≤ 5 cm	1.694 (1.175–2.441)	0.005	1.374 (1.363–2.902)	0.127
Tumor number, multiple vs. solitary	2.575 (1.569–4.226)	< 0.001	2.297 (1.214–4.349)	0.011
Resection margin, < 1 vs. ≥ 1 cm	1.978 (1.361–2.875)	< 0.001	1.089 (1.363–2.902)	0.032
Anatomical resection, yes vs. no	0.778 (0.488–1.240)	0.291		
MVI, yes vs. no	2.653 (1.829–3.846)	< 0.001	1.207 (1.085–5.760)	0.011
lymph node metastasis, yes vs. no	2.603 (1.792–3.780)	< 0.001	1.140 (1.079–1.447)	0.029
Differentiation, moderate-poor vs. well	1.689 (0.876–3.256)	0.118		
Blood loss, > 400 vs. ≤400 ml	0.772 (0.537–1.111)	0.163		
Blood transfusion, yes vs. no	1.333 (0.918–1.937)	0.131		
Operation time, > 300 min vs. ≤300 min	1.059 (0.826–1.469)	0.546		
Adjuvant chemotherapy, yes vs. no	0.830 (0.782–1.633)	0.116		
NLR^*^, > 2.96 vs. ≤2.96	2.236 (1.539–3.247)	< 0.001	1.321 (0.824–2.118)	0.248
LMR, ≤ 4.4 vs. >4.4	2.054 (1.420–2.971)	< 0.001	1.403 (0.880–2.237)	0.155
PLR, ≥ 300 vs.<300	1.931 (1.187–3.143)	0.008	1.351 (1.014–2.169)	0.017
SIS, 0	Reference			
1	2.065 (1.895–4.956)	< 0.001	1.752 (1.067–2.877)	0.027
2	3.935 (1.780–4.840)	< 0.001	2.165 (1.276–3.673)	0.004
PNI, ≤ 47 vs. > 47	2.024 (1.400-2.924)	< 0.001	1.723 (1.185–2.507)	0.004
ALBI, ≥ −2.70 vs.<−2.70	1.929 (1.284–2.898)	0.002	1.501 (1.065–3.144)	0.025
NPS, 0	Reference			
1	3.413 (1.788–6.515)	< 0.001	1.671 (1.022–3.027)	0.009
2	5.844 (3.000-11.383)	< 0.001	2.208 (1.259–4.780)	0.007

Note: ^**#**^These variables found significant at *P* < 0.1 in univariable analyses were entered into multivariable analyses. ^*^NLR, LMR SIS, PNI, and ALBI were analyzed separately with NPS (or Child-Pugh) to avoid collinearity. **Abbreviations**: ASA: Physical Status classification system; HBV: hepatitis B virus; PLT: platelet count; ALT: alanine aminotransferase; AST: aspartate aminotransferase; CEA: carcinoembryonic antigen; CA19-9: Carbohydrate Atigen19-9; MVI: microvascular invasion; NLR: neutrophil to lymphocyte ratio; LMR: the lymphocyte to monocyte ratio; PLR: platelet‑to‑lymphocyte ratio; SIS: systemic inflammation score; PNI: prognostic nutritional index; ALBI: albumin–bilirubin; NPS: Naples prognostic score; MV: multivariable; NA: not available; HR: hazard ratio; UV: univariable; NS: no significance

**Table 4:** Univariable and multivariable Cox regression analyses of prognostic factors associated with recurrence-free survival for patients with intrahepatic cholangiocarcinoma after hepatectomy

Variables	UV HR (95% CI)	UV *P*	MV HR (95% CI)	MV *P*^#^
Sex, male vs. female	1.064 (0.744–1.522)	0.733		
Age, > 65 vs.≤ 65 years	1.013 (0.712–1.442)	0.943		
Co-morbid illness, yes vs. no	1.174 (0.758–1.819)	0.473		
ASA,>2 vs. ≤ 2	1.072 (0.708–1.622)	0.744		
Performance status, ≥ 1 vs. <1	1.082 (0.759–1.543)	0.662		
HBV, yes vs. no	1.339 (0.915–1.960)	0.133		
PLT, ≥ 100*10^9^/L vs. < 100*10^9^/L	1.138 (0.555–2.331)	0.725		
ALT, > 40 vs. ≤ 40 IU/L	1.491 (0.838–2.652)	0.174		
AST, > 40 vs. ≤ 40 IU/L	1.019 (0.567–1.831)	0.950		
Child-Pugh, B vs. A	1.030 (0.660–1.609)	0.895		
Cirrhosis, yes vs. no	1.021 (1.009–1.513)	0.018		
CEA, > 10 vs. ≤10 ug/L	1.086 (0.753–1.568)	0.658		
CA19-9, > 37 vs. ≤37 ug/L	2.830 (1.968–4.070)	< 0.001	NS	
Maximum tumor size,> 5 vs. ≤ 5 cm	2.177 (1.513–3.133)	< 0.001	1.960 (1.313–2.926)	0.001
Tumor number, multiple vs. solitary	2.433 (1.454–4.070)	0.001	2.199 (1.130–4.280)	0.020
Resection margin, < 1 vs. ≥ 1 cm	1.055 (1.006–1.511)	0.017	1.012 (1.002–1.459)	0.041
Anatomical resection, yes vs. no	0.817 (0.520–1.283)	0.379		
MVI, yes vs. no	2.749 (1.896–3.987)	< 0.001	2.623 (1.430–4.809)	0.002
lymph node metastasis, yes vs. no	1.760 (1.169–2.652)	0.007	1.641 (1.125–3.116)	0.008
Differentiation, moderate-poor vs. well	1.067 (0.621–1.833)	0.813		
Blood loss, > 400 vs. ≤400 ml	1.240 (0.849–1.813)	0.266		
Blood transfusion, yes vs. no	1.085 (0.747–1.578)	0.668		
Operation time, > 180 vs. ≤180 min	1.108 (0.725–1.694)	0.634		
Adjuvant chemotherapy, yes vs. no	0.720 (0.507–1.022)	0.066	NS	
NLR^*^, > 2.96 vs. ≤2.96	1.694 (1.182–2.426)	0.004	1.255 (0.792–1.990)	0.333
LMR, ≤ 4.4 vs. >4.4	1.967 (1.375–2.813)	< 0.001	1.345 (0.851–2.125)	0.204
PLR, ≥ 300 vs.<300	1.897 (1.210–2.976)	0.005	1.718 (1.141–2.893)	0.012
SIS, 0	Reference		Reference	
1	2.291 (1.462–3.589)	< 0.001	1.459 (0.865–2.462)	0.157
2	2.645 (1.638–4.269)	< 0.001	1.655 (1.031–2.655)	0.037
PNI, ≤ 47 vs. > 47	1.853 (1.291–2.660)	0.001	1.467 (1.008–2.133)	0.045
ALBI, ≥ −2.70 vs.<−2.70	1.517 (0.991–2.322)	0.055	1.242 (0.788–1.958)	0.351
NPS, 0	Reference		Reference	
1	2.325 (1.364–3.963)	0.002	1.506 (1.184–3.498)	0.010
2	5.844 (3.000-11.383)	< 0.001	2.141 (2.519–4.087)	0.001

Note: ^#^These variables found significant at *P* < 0.1 in univariable analyses were entered into multivariable analyses. ^*^NLR, LMR SIS, PNI, and ALBI were analyzed separately with NPS (or Child-Pugh) to avoid collinearity. **Abbreviations**: ASA: Physical Status classification system; HBV: hepatitis B virus; PLT: platelet count; ALT: alanine aminotransferase; AST: aspartate aminotransferase; CEA: carcinoembryonic antigen; CA19-9: Carbohydrate Atigen19-9; MVI: microvascular invasion; NLR: neutrophil to lymphocyte ratio; LMR: the lymphocyte to monocyte ratio; PLR: platelet‑to‑lymphocyte ratio; SIS: systemic inflammation score; PNI: prognostic nutritional index; ALBI: albumin–bilirubin; NPS: Naples prognostic score; MV: multivariable; NA: not available; HR: hazard ratio; UV: univariable; NS: no significance

### Prognostic performance

**Fig. 2:**
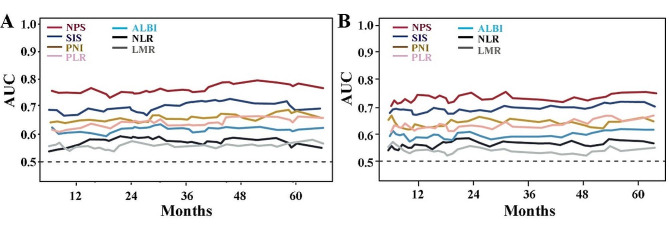
(**A**) Compared the predictive ability of postoperative overall survival by time-dependent ROCs between the NPS scores and the other indicators. (**B**) Compared the predictive ability of postoperative recurrence-free survival by time-dependent ROCs between the NPS scores and the other indicators. Abbreviations: AUCs; areas under the curves; NLR; neutrophil to lymphocyte ratio; LMR; the lymphocyte to monocyte ratio; PLR: platelet‑to‑lymphocyte ratio; SIS: systemic inflammation score; PNI: prognostic nutritional index; ALBI: albumin–bilirubin; NPS: Naples prognostic score

The time-dependent ROC curves were then performed to further discriminate which scoring system was better at predicting prognosis. The estimated AUC was calculated at different time points by the time-dependent ROC curves. The AUC of NPS, NLR, LMR, PLR, SIS, PNI and ALBI for OS were 0.753 (0.675–0.817), 0.598 (0.535–0.629), 0.554 (0.507–0.664), 0.629 (0.575–0.699), 0.705 (0.647–0.757), 0.650 (0.563–0.671), and 0.612 (0.533–0.641), respectively (Fig. 2A). According, the AUC of NPS, NLR, LMR, PLR, SIS, PNI and ALBI for RFS were 0.720 (0.619–0.784), 0.566 (0.515–0.627), 0.529 (0.507–0.601), 0.628 (0.524–0.694), 0.697 (0.568–0.757), 0.644 (0.564–0.681), and 0.560 (0.518–0.593), respectively (Fig. 2B). The comparison results of AUC between NPS and other groups indicate that the prognostic ability of NPS is significantly higher than that of other scoring systems (all *P* < 0.05).

### Subgroup analysis

**Fig. 3:**
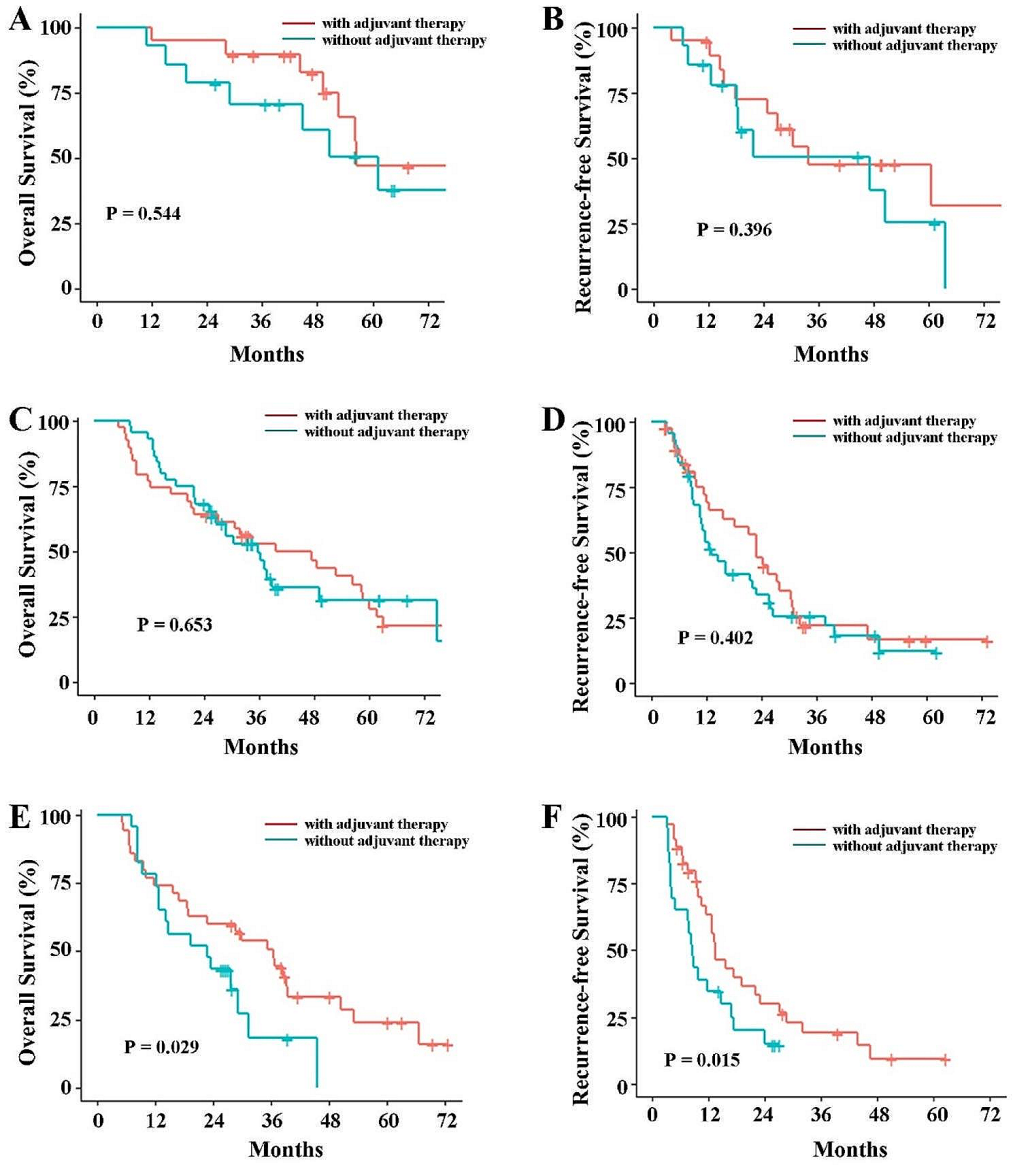
The K-M curves comparisons of overall survival and recurrence free survival between with and without adjuvant chemetherapy. (**A**) Overall survival in Group 1, (**B**) Recurrence-free survival in Group 1, (**C**) Overall survival in Group 2, (**D**) Recurrence-free survival in Group 2, (**E**) Overall survival in Group 3, (**F**) Recurrence-free survival in Group 3. Group 1 (NPS = 0); Group 2 (NPS = 1 or 2), and Group 3 (NPS = 3 or 4), respectively

To clarify the role of NPS in postoperative adjuvant chemotherapy, we further investigated the survival differences between patients who received adjuvant chemotherapy and those who did not, across different NPS values. In group 1 (NPS = 0, *n* = 33), 14 (42%) patients received adjuvant chemotherapy. And in Group 2 (NPS = 1 or 2, *n* = 83), 44 (53%) patients received adjuvant chemotherapy. Moreover, in Group 3 (NPS = 3 or 4, *n* = 58), 23 (40%) patients received adjuvant chemotherapy. The survival analysis showed that adjuvant chemotherapy did not improve OS (Fig. 3A and C) and RFS (Fig. 3B and D) in group 1 and group 2 patients, but significantly improved OS (Fig. 3E) and RFS (Fig. 3F) in group 3 patients.

## Discussion

In the present study, a total of 174 patients were included, and stratified into 3 groups based on the value of preoperative NPS. The baseline data showed that higher NPS scores were significantly associated with poorer liver function and more advanced tumors, indicating that NPS has good stratification power for patients with ICC. After hepatectomy, it was found in the comparison of perioperative complications that the higher the NPS, the higher the overall complication rate (*P* = 0.048), especially for minor morbidity (*P* = 0.026). However, there was no statistical difference in the incidence of major complications (*P* = 0.841), which may be related to the low incidence of major complications. In the multivariable Cox regression analysis, it was observed that NPS serves as an independent predictor for both OS and RFS. In other words, patients in group 2 exhibited a nearly 1.5-fold higher risk of tumor recurrence and death compared to those in group 1. Similarly, patients in group 3 faced a nearly 2-fold increased risk of tumor recurrence and death. Furthermore, when compared to other previously reported prognostic models, the NPS displayed superior discriminatory and predictive capabilities. In addition, the results showed that for the entire cohort, there was no survival benefit from adjuvant chemotherapy. However, patients with high NPS may benefit from adjuvant chemotherapy. Findings from this study contribute to an emerging body of evidence elucidating the clinical value of NPS in predicting outcomes for patients with ICC after hepatectomy.

Chronic inflammation is associated with the development of various tumors, especially for ICC [25–27]. Chronic inflammation enhances cholangiocyte exposure to inflammatory mediators, leading to the accumulation of mutations in tumor proto-oncogenes, suppressor genes, and DNA mismatch-repair genes. Moreover, chronic inflammation creates a permissive environment that promotes tumor growth, metastasis, and chemoresistance. The prognostic significance of the inflammation on ICC has been stressed but was insufficiently taken into account in the search for classifications of ICC adapted to clinical treatment. The stratification of ICC patients into subgroups based on their inflammatory status and clinical features would facilitate more effective disease management [28]. Peripheral blood cell counts, including white blood cells, neutrophils, and lymphocytes, can serve as indicators of a patient’s inflammatory status. Previous research has shown that a high density of tumor-infiltrating lymphocytes is associated with improved prognosis. Neutrophils possess the ability to produce cytokines, which can stimulate tumor angiogenesis and cancer cell proliferation. Additionally, these cells also contribute to the suppression of lymphocyte-mediated cytolysis. Furthermore, it has been reported that elevated platelet counts are linked to a pro-tumorigenic environment.

Increasing evidence has demonstrated that serum inflammatory indicators, such as NLR, LMR, SIS, and PLR are associated with the prognosis of ICC [9–11]. Ohira et al. reported, based on 52 patients with ICC, that NLR, LMR, and SIS were independently associated with poor survival, but PLR was not [29]. In addition, Wu et al. performed a retrospective study based on 123 patients with ICC, and the results showed that NLR and LMR were significantly associated with OS [9]. Meanwhile, Chen et al. evaluated the prognostic significance of PLR based on 322 patients with ICC. The results showed that PLR represents an independent adverse prognostic factor for OS and RFS in patients with ICC [10]. However, Zhang et al. compared the prognostic value of different inflammatory indicators, and the results showed that SIS had higher prognostic efficiency than LMR, NLR, and PLR. Moreover, NLR and LMR were only significantly associated with OS, but not significantly related to time to recurrence [11]. In the present study, the results also showed that SIS and PLR are significantly associated with survival, but LMR and NLR were not. Through the above study findings, it is found that there are significant differences in the results of different studies. The possible reason is that the study failed to deal with the colinearity, making the conclusion less robust. Therefore, combining similar variables into one integrated variable as much as possible is the main solution to reducing colinearity and improving prediction performance.

Nutrition is another important indicator that affects the prognosis of cancer patients, which is also recognized in ICC. The liver is the main site of nutrient metabolism and synthesis, but chronic hepatitis often leads to damage to the liver’s ability to synthesize nutrients. Therefore, there exists a significant correlation between nutrition, inflammation, and tumor development. Serum albumin levels, which serve as a measure of preoperative nutritional status, have traditionally been considered one of the most significant prognostic indicators among patients undergoing cancer surgery. Decreased serum albumin expression has been recognized as a marker of malnutrition and a weak immune defense system, and its substantial reduction frequently serves as a warning sign of postoperative complications for clinicians. Tsilimigras et al. reported that the ALBI score was associated with both short- and long-term outcomes following resection for ICC [15]. In the present study, ALBI was significantly associated with OS but not RFS. The potential reason is that ALBI can effectively reflect liver function, but cannot reflect inflammatory level. Previous studies also have attempted to predict the prognosis of patients with ICC by combining inflammatory and nutritional indicators, but have not been able to integrate these indicators into a single variable [30, 31]. Akgül et al. conducted a retrospective study aiming to evaluate the value of PNI, an index that combines serum albumin and lymphocyte count, in the prognosis of patients with ICC [14]. The results showed that PNI was associated with a more aggressive ICC phenotype and a markedly worse prognosis. In the present study, PNI was also significantly associated with OS and RFS. However, the predictive performance of PNI is not high, with a median AUC of only about 0.65. The potential reason may be that serum albumin and lymphocytes cannot comprehensively translate the nutritional and inflammatory status. Therefore, the current study first explored the relationship between the prognosis of ICC and NPS, a new comprehensive index of both the nutritional and immunologic status. The results of the study confirm that NPS is significantly associated with long-term prognosis in patients with ICC, and is significantly superior to other scoring systems in predicting the prognosis. What’s more, subgroup analysis showed that patients with high NPS values could benefit from postoperative adjuvant chemotherapy, suggesting that NPS has the value of guiding postoperative adjuvant treatment.

This study also has some limitations. Firstly, although the AUC value (0.753 for OS and 0.720 for RFS) is not as high as desired, it is only evaluating the impact of one variable on prognosis. To achieve more accurate predictions, it is essential to consider additional independent risk factors in combination. Secondly, although patients in the high NPS group may benefit from postoperative adjuvant chemotherapy, large-scale multi-center studies are still needed due to the low sample size. Thirdly, in order to better clarify the predictive value of NPS, this study excluded patients who relapsed within 30 days and died within 90 days. These patients had poor prognosis, which was more likely caused by advanced tumor stage, although NPS might have played a role in this process. Fourthly, as a retrospective study, there is an inherent bias, as variables that could not be standardized or identified, as well as patients lost to follow-up, may exist. Therefore, further validation, especially through multicenter RCTs, is still necessary.

## Conclusion

To conclude, the NPS has been suggested as an easily accessible and measurable biomarker that integrates both inflammation and nutrition. The present study suggests that the preoperative NPS value is a unique and independent predictor for predicting a poor prognosis and recurrence in patients with ICC who underwent curative resection. The preoperative prediction of prognosis using the NPS could potentially be utilized to guide pre- and postoperative therapies aimed at enhancing patient outcomes.

### Electronic supplementary material

Below is the link to the electronic supplementary material.

Supplementary Material 1

## Data Availability

The datasets used and analyzed during the current study are available from the corresponding author upon reasonable request.

## Abbreviations

NPS: Naples prognostic score
ICC: intrahepatic cholangiocarcinoma
HBV: hepatitis B virus
NLR: neutrophil to lymphocyte ratio
LMR: the lymphocyte to monocyte ratio
PLR: platelet‑to‑lymphocyte ratio
SIS: systemic inflammation score
PNI: prognostic nutritional index
ALBI: albumin–bilirubin
ASA: physical Status classification system
PLT: platelet count
ALT: alanine aminotransferase
AST: aspartate aminotransferase
CEA: carcinoembryonic antigen
CA19-9: Carbohydrate Atigen19-9
MVI: microvascular invasion
PHLF: post hepatectomy liver failure
MV: multivariable
UV: univariable
HR: hazard ratio
CI: confidence interval
