# Supplementary figures and images for: Prognostic value of the Naples prognostic score in patients with intrahepatic cholangiocarcinoma after hepatectomy
Source: BMC Cancer. 2024 Jun 14;24:727. doi: 10.1186/s12885-024-12502-4 (PMC11177390; doi:10.1186/s12885-024-12502-4)

**Supplement Figure 1.** Calculation of the Naples Prognostic Score.


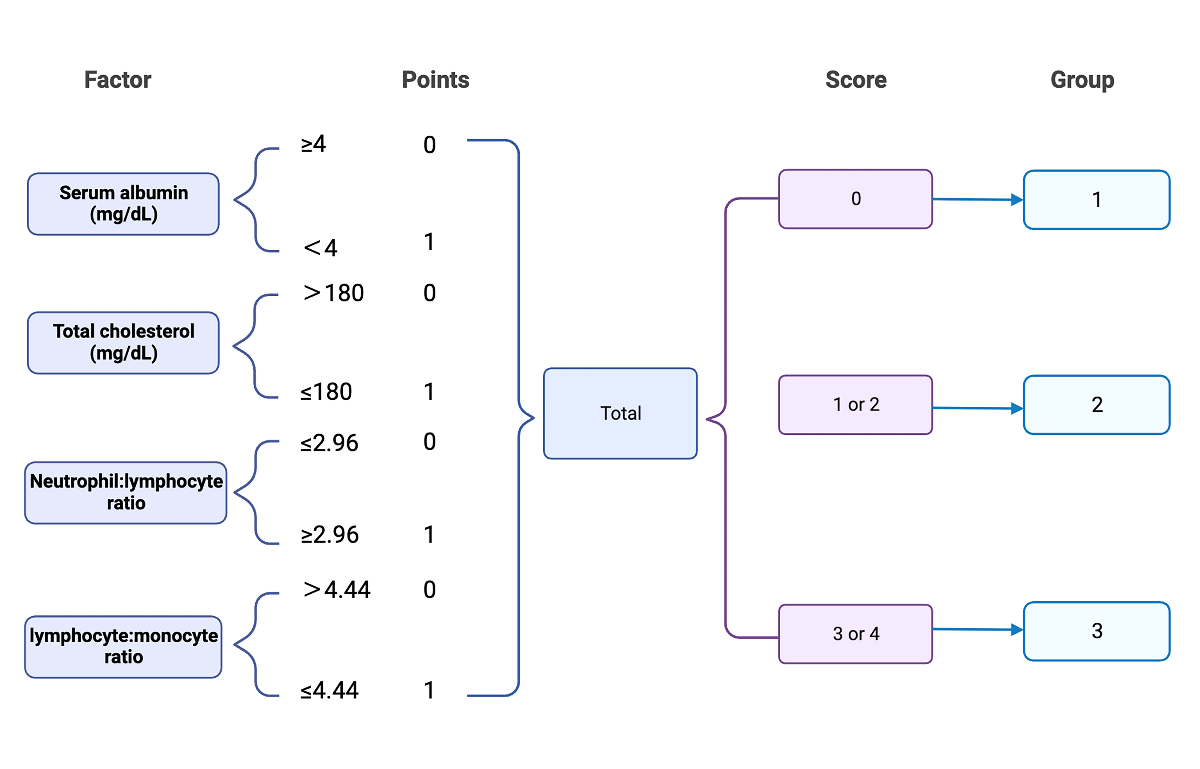

Supplement: Supplementary file 1 — Supplementary Material 1 [file 12885_2024_12502_MOESM1_ESM.docx]
